# C_2_N: A Class of Covalent Frameworks with Unique Properties

**DOI:** 10.1002/advs.202001767

**Published:** 2020-11-13

**Authors:** Zhihong Tian, Nieves López‐Salas, Chuntai Liu, Tianxi Liu, Markus Antonietti

**Affiliations:** ^1^ Key Laboratory of Materials Processing and Mold (Zhengzhou University) Ministry of Education National Engineering Research Center for Advanced Polymer Processing Technology Zhengzhou University Zhengzhou Henan 450002 China; ^2^ Department of Colloid Chemistry Max Planck Institute of Colloids and Interfaces Potsdam 14476 Germany; ^3^ Key Laboratory of Synthetic and Biological Colloids Ministry of Education School of Chemical and Material Engineering Jiangnan University Wuxi 214122 P. R. China

**Keywords:** applications, C_2_N, carbon materials, heteroatoms, regular pores

## Abstract

C_2_N is a unique member of the C_n_N_m_ family (carbon nitrides), i.e., having a covalent structure that is ideally composed of carbon and nitrogen with only 33 mol% of nitrogen. C_2_N, with a stable composition, can easily be prepared using a number of precursors. Moreover, it is currently gaining extensive interest owing to its high polarity and good thermal and chemical stability, complementing carbon as well as classical carbon nitride (C_3_N_4_) in various applications, such as catalysis, environmental science, energy storage, and biotechnology. In this review, a comprehensive overview on C_2_N is provided; starting with its preparation methods, followed by a fundamental understanding of structure–property relationships, and finally introducing its application in gas sorption and separation technologies, as supercapacitor and battery electrodes, and in catalytic and biological processes. The review with an outlook on current research questions and future possibilities and extensions based on these material concepts is ended.

## Graphical abstract

This review gives an overview on unique structural characteristics of C_2_N including its noble character, multipodal binding sites, and high hydrophilicity. The structure–property relationships and performance of C_2_N for different applications are reviewed. The future perspectives of the field are discussed at the end to highlight the possibility of using this new material‐ in various applications.

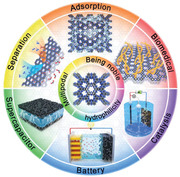

## 1 Introduction

The exploration of novel materials, from bronze over iron to plastic and silicon, and understanding their structure–property relations has always been a driving factor for economic, social, and cultural development. Currently, a new aspect required in this research is the search for sustainability, i.e., the materials should be prepared using omnipresent and cyclical resources. Moreover, the tasks are becoming highly functional and complex; hence, “little” is now considered to be “more.”

A scientific increase in the use of not only functional carbon materials, including graphene, carbon nanotubes, or fullerenes, but also carbon nitride (CN) frameworks is expected owing to their unique electronic features and chemical/physical properties that are promising for key applications across the fields of chemistry, physics, biology, and medicine.^[^ ^1^ ^]^

Moreover, the covalent binding of C and N in regular structural patterns seems to be extremely stable, and the surge to discover such useful materials has led to the development of an entire group of CN materials such as C_2_N,^[^ ^2^, ^3^ ^]^ C_3_N,^[^ ^4^, ^5^, ^6^, ^7^ ^]^ C_4_N,^[^ ^8^, ^9^, ^10^ ^]^ C_3_N_2_,^[^ ^11^ ^]^ C_3_N_4_,^[^ ^12^, ^13^, ^14^, ^15^ ^]^ C_3_N_5_,^[^ ^16^, ^17^, ^18^ ^]^ C_3_N_6_,^[^ ^19^ ^]^ and C_4_N_2_.^[^ ^20^ ^]^ These CN materials can be prepared by simple polycondensation of various organic molecular monomers, and their optical, electronic properties, and structural parameters could be easily controlled by simply modifying the synthetic procedures or hybridizing with other nanostructures. Among these CNs, the most studied C_3_N_4_ exhibits semiconducting properties and has gained significant importance in a wide range of application possibilities such as heterogeneous photocatalysis,^[^ ^12^ ^]^ water molecules actuators,^[^ ^21^, ^22^ ^]^ optoelectronic sensing,^[^ ^23^, ^24^, ^25^ ^]^ biosensing/bioimaging,^[^ ^26^, ^27^ ^]^ and photoelectrochemical conversion.^[^ ^28^, ^29^ ^]^ The successful dissolution of C_3_N_4_ allowed the homogeneous CN photocatalyst.^[^ ^30^, ^31^, ^32^ ^]^ Its catalytic activity is 10 times higher than that of the heterogeneous counterpart. Thus, it is expected that it will be able to bridge the gap between homogeneous and heterogeneous catalysis. In addition, C_3_N_4_ also could act as template for synthesizing refined carbon nanostructures since it is usually stable up to 550 °C and undergoes complete thermolysis at 750 °C.^[^ ^33^ ^]^ For further discussion about the advances of C_3_N_4_ materials, we refer the reader to topical reviews by Liu,^[^ ^34^ ^]^ Vinu,^[^ ^35^ ^]^ and Zhang.^[^ ^36^ ^]^ C_2_N materials arisen in recent years are stable up to 700–750 °C.^[^ ^2^, ^3^ ^]^ Compared with C_3_N_4_, C_2_N has higher specific surface area, thermal stability and conductivity. The unique electronic properties of C_2_N could be complementary to the use of C_3_N_4_ in application areas such as adsorption,^[^ ^37^, ^38^ ^]^ electrochemical catalysis and energy storage.^[^ ^39^, ^40^ ^]^

**Figure 1:**
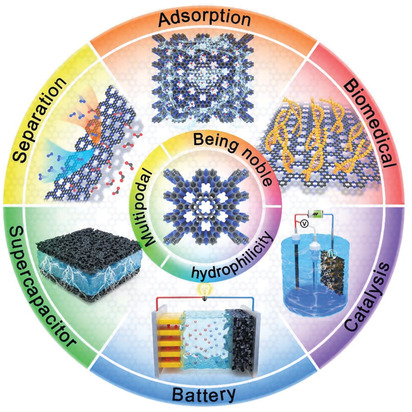
Schematic illustration of structure, unique features, and wide applications of C_2_N materials.

The focus structure of the current review, C_2_N, has a remarkably well‐defined skeleton and structural nanopores (**Figure** **1**). Moreover, C_2_N has a graphene‐like structure in which 1/3 of all the carbon atoms are replaced with pyrazinic nitrogen atoms. This results in the C_2_N having a covalent “zeolite‐like” structure where the pores are regular, ideally with a 12‐atom sized circumvent. The unique porous structure of the C_2_N in which benzene rings are connected by bridging the nitrogen atoms produces a large *π* electron pool in the benzene rings and generates intrinsic electron density (partially negative) on the N atoms.^[^ ^2^ ^]^ This introduction of N atoms into the carbon lattice enhances the properties of carbon materials such as chemical and thermal stability, band positions, catalytic efficiency, and oxidation stability^[^ ^41^ ^]^ and contributes to specific interactions with liquids, solvated ions, or gases.^[^ ^42^, ^43^ ^]^

Owing to the predominant paracharacter of the substitution patterns, all doping centers could easily communicate with each other electronically. Therefore, when compared to other CN materials, the significance of C_2_N arises from the fact that the heteroatoms are in a particularly attractive mutual arrangement, and thus, the properties of the collective framework are altered by the concerted action of at least two doping atoms that are jointly active via the para conjugation. We have discussed the influence of this pattern to explain the special performances in gas/water adsorption,^[^ ^37^, ^38^ ^]^ catalysis,^[^ ^44^ ^]^ and energy storage.^[^ ^40^, ^45^, ^46^, ^47^, ^48^ ^]^ Furthermore, the pores with six regularly spaced nitrogen lone pairs in the lining ring (N6‐cavities) are extremely suitable for anchoring metallic nanoparticles and single atoms, acting as an “active support.”^[^ ^39^, ^40^, ^41^, ^42^, ^43^, ^44^, ^45^, ^46^, ^47^, ^48^, ^49^, ^50^, ^51^ ^]^

In this review, we cover the recent advances in the synthesis of C_2_N, the understanding of its structure–property relations, and summarize the wide‐ranging applications that extend from environment‐ and energy‐related fields (e.g., CO_2_ adsorption, batteries, catalysis) to various emerging fields (e.g., biomedical technologies). In addition, the current state of the art of C_2_N materials and our view on future research are also discussed in this review.

## 2 Synthetic Routes for C_2_N

A key factor to form a well‐defined C_2_N is the selection of the right monomers, which are able to prealign and “click” into new directional covalent bonds. Hence, having the knowledge of building units and synthetic organic routes (i.e., reaction media and synthetic conditions) is a prerequisite. C_2_N can be prepared using common methods, including solvothermal, ionothermal, and direct bulk condensation; however, its porosity, functional edge termination, and structural regularity might change. The solvothermal method at low temperatures fosters the formation of a regular 2D crystalline structure, which is expected to widen the bandgap to an ideal level for semiconductor applications.^[^ ^52^, ^53^ ^]^ The ionothermal and direct bulk condensation at higher temperatures not only promotes the formation of planes, but also rings and further condensation between the pore walls. As a result, a 3D highly porous structure, which can provide more active sites and space for guest molecules is obtained. Such structures are very appealing in the field of adsorption and energy storage. In this review, we have summarized the progress achieved in the preparation of C_2_N using different synthetic routes.

### 2.1 Wet‐Chemical Routes

**Figure 2:**
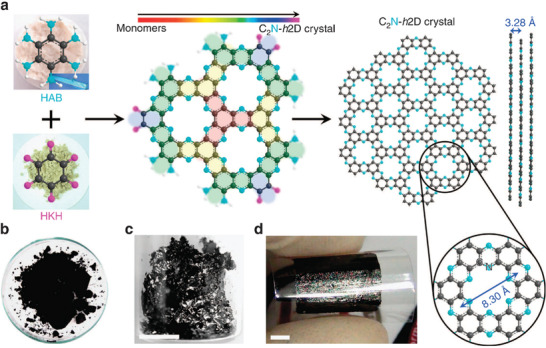
Preparation and structure of C_2_N‐*h*2D crystal. a) Schematic representation of the reaction between hexaaminobenzene (HAB) trihydrochloride and hexaketocyclohexane (HKH) octahydrate to produce the C_2_N‐*h*2D crystal; the inset in the image of HAB is a polarized optical microscopic image of a single HAB crystal. Digital photographs: b) as‐prepared C_2_N‐*h*2D crystal; c) solution‐cast C_2_N‐*h*2D crystal on SiO_2_surface after heat‐treatment at 700 °C; d) a C_2_N‐*h*2D crystal film (thickness: ≈330 nm) transferred onto a PET substrate. The shiny metallic reflection of the sample indicates that it is highly conductive. Reproduced with permission.^[^ ^2^ ^]^ Copyright 2015, Springer Nature.

Solvents, monomers, and catalysts are mixed and reacted for a certain time at the specified temperature. The resulting condensed precipitate is collected, washed, and dried to produce C_2_N as a solid powder. Mahmood et al. synthesized the first C_2_N‐*h*2D using this method (**Figure** **2**). In this approach, hexaaminobenzene (HAB) trihydrochloride and hexaketocyclohexane (HKH) octahydrate were condensed in N‐methyl‐2‐pyrrolidone in the presence of a few drops of sulfuric acid or trifluoromethanesulfonic acid.^[^ ^2^ ^]^ The huge potential energy obtained by aromatization is the reason for the spontaneous polycondensation between HAB and HKH and leads to the formation of a layered crystalline 2D structure.^[^ ^54^ ^]^ Using such a powerful driving force for aromatization, the 3D fused *π*‐conjugated microporous polymers can be conveniently achieved by ionothermal processes in the presence of AlCl_3_.^[^ ^55^ ^]^ The obtained 2D crystal C_2_N‐*h2*D is a semiconductor, and the bandgap was determined as 1.96 eV. The on/off current ratio of a field‐effect transistor formed using this material attained an incredible value of 10^7^. This work indeed presented a highly efficient synthesis of layered 2D holey crystals via a simple bottom‐up wet‐chemical approach without template assistance. This powerful method coupled with the versatility of organic reactions can be extended to synthesize similar 2D materials with adjustable properties.

### 2.2 Salt Melt Synthesis

**Figure 3:**
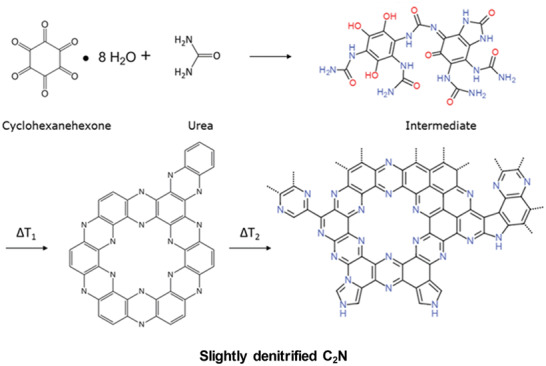
Schematic representation of synthesis based on cyclohexanehexone and urea reactants to a cross‐linked intermediate material (top), which systematically converts to a disordered C_2_N at temperatures greater than 500 °C (*T*_1_<*T*_2_), then to a slightly nitrogen‐depleted C_2_N containing primarily pyridinic and pyrazinic nitrogen (bottom). Reproduced with permission.^[^ ^3^ ^]^ Copyright 2016, Wiley‐VCH.

In recent years, salt melt synthesis, which uses molten inorganic salts as the solvent, has gained extensive attention in the field of carbon material synthesis, and has become another research hotspot.^[^ ^56^, ^57^ ^]^ The molten salts are more thermally stable (up to 1000 °C) and have lower viscosity as compared to those of water and other organic solvents. Functional carbon materials with high specific surface area and adjustable pores were prepared using the salt melt method, and the pore structure was regulated by adjusting the type and proportion of salt. This method was extended to produce “C_2_N”‐species, while being able to specifically control size and functionality of the superstructure of the condensate. For example, Fechler et al. proposed a method for synthesizing a bulk C_2_N structure by a salt melting reaction using deep eutectic mixtures composed of hexaketocyclohexane and urea (**Figure** **3**).^[^ ^3^ ^]^ Simple urea can be used as an effective nitrogen source and melting point depressant. Due to the strong hydrogen bonding, hexaketocyclohexane and urea form a deep eutectic liquid mixture, which is very suitable for liquid‐based carbon synthesis. In addition, various phenol/ketone precursors can be chosen to control the initial orientation and degree of cocondensation of the final material, thereby controlling the nanostructure, functionality, and heteroatom‐doping. The obtained C_2_N products exhibited low long‐range order, i.e., they were not crystalline. However, they maintained an extremely well‐defined micropore structure at a high density, and the pores were tightly covered by pyrazinic nitrogen. The fact that the complicated hexaaminobenzene could be replaced by urea indicated the high stability of the final structure, i.e., The structure of C_2_N is in a thermodynamic minimum state that resulted in similar products even for extremely different reactions. The micropores of this polymer structure were practically indistinguishable from the ideal C_2_N.

Salt melt synthesis is a relatively green process as many of the salts used are not volatile or harmful.^[^ ^56^ ^]^ In other words, they are typically environmentally benign and can be easily recycled. As a result, the industrialization of this process could be beneficial.

### 2.3 Direct Condensation

The direct condensation of monomers uses a bulk method that does not require a solvent/salt melt medium or template. It is easy to operate and relatively easier to scale‐up, but it is extremely demanding sterically. The structures of the materials produced using this method depend entirely on the primary crystal size and orientation of the precursors in the crystal. Walczak et al. reported the synthesis of porous covalent materials with a stoichiometry close to that of C_2_N by direct thermal annealing of the hexaazatriphenylene (HAT) crystals as precursors.^[^ ^38^ ^]^ Although no porogen or metal species are involved in the synthetic process, the specific surface areas of the final materials still reached up to 1000 m^2^ g^−1^, which is approximately the expected value for that of the microporosity of C_2_N. HAT is an electron deficient, planar, rigid, and aromatic discotic system with excellent *π*–*π* stacking ability and by itself exhibits remarkable oxidation resistance.^[^ ^58^, ^59^ ^]^ In particular, its strong supramolecular aggregation and ability to self‐assemble into crystalline structures makes it a promising material for the bulk synthesis of C_2_N. Note that, pristine HAT is already fully sp‐ or sp^2^‐conjugated and it does not contain even a single hydrogen atom. Therefore, C_2_N could be formed without any structural rearrangements, using only electrocyclic additions and eliminations. The elimination product in this case is dicyan, an extremely stable gas. The content and type of nitrogen species in the final condensation product can be controlled with the carbonization temperature, while the porosity stays largely unaffected. This molecular precursor method opens new possibilities for the preparation of porous noble carbons with high molecular control, and provides a way to access the special physical properties of the C_2_N structure. However, this method synthesizes less ordered polymer‐type species.

### 2.4 Doping of C_2_N

The substitution of foreign atoms could affect the structural and electronic properties of C_2_N. Heteroatom‐doped C_2_N can be prepared by the method described above. Shinde et al. introduced a two‐step strategy for the construction of porous 3D sulfur‐doped holey C_2_N aerogels (S‐C_2_N).^[^ ^48^ ^]^ First, chloroanilic acid was aminated with ethylenediamine to form an intermediate hexaaminobenzene complex. The as‐prepared hexaaminobenzene was then polymerized in the presence of chloroanilic acid, l‐alanine, and l‐cysteine using a typical wet‐chemical reaction with 1‐methyl‐2‐pyrrolidinone as the solvent to construct *π*‐conjugated polymeric extended C–N structures. After freeze‐drying and subsequent pyrolysis, the resulting products formed an array of bifunctional, hierarchical, and porous S‐C_2_N aerogels. The sponge‐like porous S‐C_2_N aerogels had extremely low mass densities and different sizes.

To design new functionalization patterns with easy‐to‐bridge heterocycles with two heteroatoms in the p‐position, such as oxazines, dibenzodioxines, or thiazines/thiazoles, we recently used simpler, more available, and sustainable starting synthons, e.g., gallic acid, urea, and thiourea. As a result, we produced 1,4‐para N,O‐dual doped C_2_N*_x_*O_1‐_ *_x_* and tri‐doped C_2_(N*_x_*O*_y_*S*_z_*)_1_ materials using similar salt melt synthesis and ring closure methods.^[^ ^37^, ^40^ ^]^ Gallic acid is considered to be a promising unit for ordered structure synthesis because it can easily decarboxylate at higher temperatures to generate pyrogallol. Therefore, the carboxylic acid unit can be regarded as a “protecting/leaving group” that controls preorganization, functionality, and N/O/S doping in the target carbon material. The prepared multifunctional materials exhibited an unusually high N/O or N/O/S heteroatom content (just below the ideal value of 33 mol%) and high specific surface area with defined microporosity, the size and shape of which were controlled by the condensed chemical structure. The doping of N/O or N/O/S in the carbon lattice provided several remarkable characteristics to the carbon‐based host framework, such as high thermal and oxidation stability, conductivity and strong adsorption behavior to guest molecules (e.g., CO_2_ and electrolyte ions). Herein, it is important to point out that, compared to HAB and HKH as monomers for synthesizing C_2_N, gallic acid, urea, and thiourea have the advantages of being readily available raw materials, have a low cost, and the easiness of large‐scale industrial production. Since doped C_2_N maintains the C_2_N configuration, both of them are referred to as C_2_N from here on.

## 3 Intrinsic Features of C_2_N

### 3.1 Being Noble

**Figure 4:**
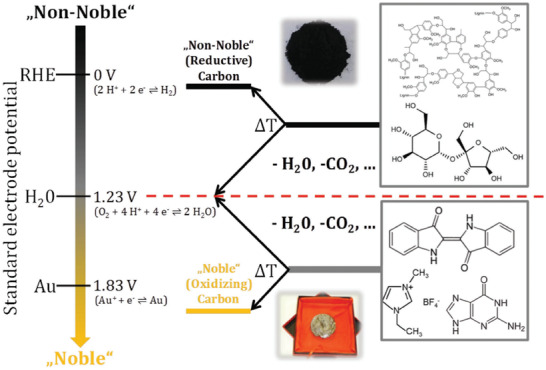
Two reaction paths of carbonization towards noble carbons (starting from barely oxidizable precursors) and non‐noble carbons (starting from easily oxidizable precursors) displayed on an electrochemical scale. Elimination of the same classical fragment drives the reactions to two different sides of the “nobility equator” (red line); either non‐noble or noble. Reproduced with permission.^[^ ^41^ ^]^ Copyright 2018, Wiley‐VCH.

As delineated in previous research,^[^ ^41^ ^]^ the key factor to consider in the preparation of noble covalent materials is the selection of the carbon precursor to be carbonized (**Figure** **4**). Assuming the typical compounds to be laminated (CO_2_, H_2_O, or NH_3_), as shown in Figure 4, there is an “equator” producing the different “carbonized” products with electrochemical properties that depends on whether the precursor selected belongs above or below the electrochemical separation line. If cellulose or plant biomass are chosen as a starting product (upper part of Figure 4), then essentially water (+1.23 V) and CO_2_ are eliminated; thus, the obtained sugar coal moves upwards in the electrochemical potential scale, i.e., it becomes reductive. This sets the base for extensive processes, such as carbothermal reduction of iron ore. However, when condensing more stable starting monomers, such as nucleobases, dopamine, or ionic liquids, the eliminated products are water, CO_2_, CO, NH_3_, or N_2_. Since these elimination products are more electrochemically negative than the starting precursors, the internal energy of the condensate reduces, moving downwards on the electrochemical potential scale. Subsequently, a truly noble carbon, carbon nitride, or a similar covalent material, which contains appropriate heteroatom motifs, is produced. In this condensation process, a majority of bonding disorder is thermodynamically eliminated, and only the most stable bonds and organized covalent structures are formed.

“C_2_N”‐species are the most recently developed microporous noble carbon materials with high specific surface area that precisely follow this concept. Regular doping of pyrazinic nitrogen makes the C_2_N materials more “noble” than pristine carbon, i.e., they oxidize substances instead of being oxidized themselves due to the highly positive working potential of their electrons.^[^ ^41^ ^]^ Owing to its high thermal, chemical and oxidative stability, C_2_N is a promising candidate to be used as a support for metal catalysts and energy storage applications, and has advantages especially over non‐noble carbon materials obtained due to the carbonization of sugar.

### 3.2 Multipodal Binding Sites

Introducing heteroatoms into covalent sp^2^‐conjugated materials makes the electronic system more polarizable, and thereby more polar. In addition to the electrons being more moveable, the introduction of different amounts of heteroatoms changes the work‐function of electrons, making the structures electron accepting or electron donating, i.e., “acidic” or “basic.”^[^ ^60^, ^61^ ^]^ Moreover, the heteroatoms with lone pairs provide specific ligand‐like binding sites for metal centers. In the case of C_2_N, the framework is composed of a line‐up of six regular nitrogen atom cavities (N6‐cavities). In 2016, Du et al. had postulated, based on modeling work, the ability of such N6‐cavities of a C_2_N monolayer to stabilize transition metal atoms (except Mn and Co atoms), using the first principle method.^[^ ^62^ ^]^ They also reported that Fe single atoms were strongly adsorbed (adsorption energy of 4.55 eV), and an energetic diffusion barrier of more than 3.00 eV was required to leave the N6‐cavities. Thus, the atoms were stable.^[^ ^63^ ^]^ In the same year, Li et al. also reported that the stabilization and energy barrier were high not only for one metal atom, but also for two atoms of transition metals in the cavities.^[^ ^64^ ^]^ However, as a general comment we have to add that a planar sixfold coordination is unusual for most traditional metal centers, and that new electronic properties as well as stabilization of unusual oxidation states from this mismatch of symmetry is expected.

In 2018, Bhattacharyya et al. reported a strong interaction of some molecules (HF, HCN, and H_2_S) with C_2_N that was observed when modelling the system using the periodic density functional theory and ab initio molecular dynamics simulations.^[^ ^65^ ^]^ Su et al. reported that certain volatile organic compounds (H_2_CO, C_6_H_6_, and C_2_HCl_3_) had stronger bonds with Al‐modified C_2_N.^[^ ^66^ ^]^ These results indicated that not only single atoms can be strongly trapped in the N6‐nanopores of C_2_N and exist in the isolated form. Moreover, the highly polarized pore‐metal hybrid can also improve the adsorption and mass transfer of reactants.^[^ ^51^ ^]^

Several theoretical predictions on the ability of C_2_N and metal composites to act as catalysts for diverse applications followed (e.g., HCOOH dehydrogenation, seawater desalination, and photocatalytic and electrocatalytic water splitting activity).^[^ ^67^, ^68^, ^69^, ^70^, ^71^, ^72^, ^73^, ^74^, ^75^, ^76^, ^77^ ^]^ The predicted multipodal strong binding sites^[^ ^66^ ^]^ play an important role when C_2_N combines with CO_2_, ionic liquids, and metal ions, such that C_2_N exhibits excellent gas adsorption/separation, supercapacitor, and battery performance,^[^ ^38^, ^40^ ^]^ which is discussed in detail in subsequent sections. Moreover, due to these unique pores, C_2_N itself can be used as a catalyst as well as a support, which can strongly bind large amounts of single metal atoms. The large corpus of predictive data obtained from theoretical modelling provided important information to material chemists to perform more sensible experiments to outperform the current benchmark materials used in various applications using C_2_N.^[^ ^78^, ^79^, ^80^, ^81^, ^82^, ^83^, ^84^, ^85^, ^86^, ^87^, ^88^, ^89^, ^90^, ^91^, ^92^, ^93^, ^94^, ^95^, ^96^, ^97^, ^98^, ^99^, ^100^ ^]^

### 3.3 High Hydrophilicity

The hydrophilicity of C_2_N strongly contributed to the performance of materials in several applications such as deionization, membrane separation, or (electro) catalysis.^[^ ^101^, ^102^, ^103^, ^104^ ^]^ In all these applications, the wettability of the carbon surface and structure of the water at the surface are fundamental aspects to evaluate their performance. The most thermodynamically stable polymorph of carbon is graphite. It is composed of some edges, but is mostly composed of aromatic sp^2^‐hybridized carbon atom planes, which makes it rather hydrophobic. A prerequisite for several graphite or graphene applications is the availability of a large density of processable delaminated sheets. Typically, the aid of excessive amounts of dispersing agents are necessary for this.^[^ ^105^ ^]^

The hydrophobicity of sp^2^‐conjugated carbons can be easily modulated by surface oxidation (e.g., functionalization with epoxy, hydroxyl, and keto groups).^[^ ^106^ ^]^ However, such a treatment of the carbon surface results in certain undesirable collateral damages. For instance, changes in structural bonds of carbon, which directly affect some fundamental properties of carbon such as conductivity or thermal stability. An alternative to postoxidation is the introduction of heteroatoms into the carbon lattice. This resulted in a change in carbon polarizability, dipole moments, and the macroscopic polarity of the solid‐state material.^[^ ^41^ ^]^

**Figure 5:**
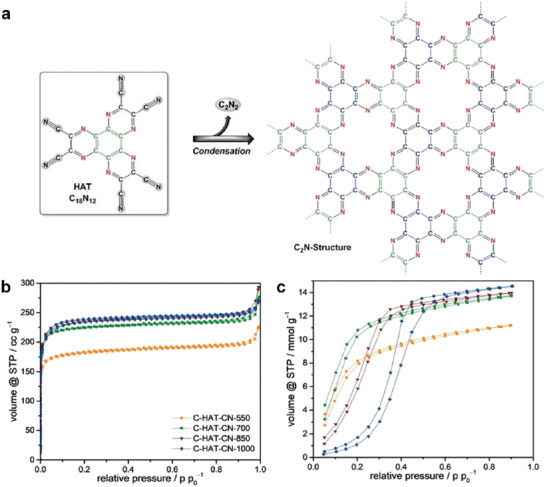
a) Idealized model for the formation of C_2_N structure by condensation of HAT‐CN precursor, b) argon physisorption isotherms (87 K), and c) water vapor physisorption isotherms (298 K) of C‐HAT materials. Reproduced with permission.^[^ ^38^ ^]^ Copyright 2018, Wiley‐VCH.

A high nitrogen doping level in combination with regular microporosity of C_2_N materials results in a strong interaction with H_2_O molecules, and its water adsorption properties are comparable to those of zeolites. For instance, the HAT‐CN materials prepared using the direct carbonization method have a uniform doping of nitrogen (**Figure** **5**a).^[^ ^38^ ^]^ The specific surface area of these materials can reach 1000 m^2^ g^−1^ (Figure 5b), and they have extremely high H_2_O adsorption capacity even at low relative pressures below *P*/*P*_0_ = 0.2 (Figure 5c). CHAT‐CN‐550 and CHAT‐CN‐700 (i.e., the materials condensed at 550 °C and 700 °C) exhibited type I H_2_O sorption isotherms with steep increases at low relative pressure, which indicated that C_2_N materials have high affinity to water owing to their zeolite‐like micropores. According to our experience, the limit of thermal stability of materials with a C_2_N stoichiometry is ≈700–750 °C.^[^ ^37^ ^]^ When the condensation temperature is increased above this limit to 1000 °C, the nitrogen content decreases and the N6‐pores are destroyed. Subsequently, the onset points of water adsorption for CHAT‐CN‐1000 shift to higher relative pressures and exhibit hysteresis. The complete resulting curves then indicate that the water adsorption mechanism resembles that of conventional N‐doped carbon with lesser polar sites.

**Figure 6:**
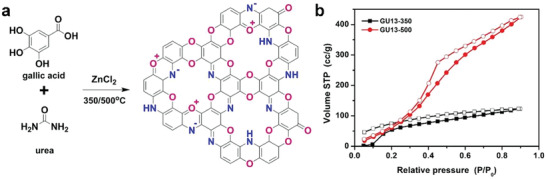
a) Idealized structure of C_2_N*_x_*O_1‐_ *_x_*. b) Water vapor physisorption isotherms at 298 K. Reproduced with permission.^[^ ^37^ ^]^ Copyright 2018,The Royal Society of Chemistry.

This unusually high hydrophilicity is also exhibited by O‐substituted C_2_N materials, C_2_N*_x_*O_1‐_ *_x_* prepared from gallic acid (G) and urea (U) as shown in **Figure** **6**.^[^ ^37^ ^]^ Similarly as for the HAT‐based systems, the regular pore system developed only when the temperature was increased up to 500 °C. For instance, the specific surface area of the sample prepared at 350 °C (i.e., GU13‐350) is only 17 m² g^−1^, while that prepared at 500 °C (i.e., GU13‐500) is as high as 1227 m² g^−1^. However, both the water vapor adsorption isotherms at 298 K show significant uptake of water even at extremely low relative pressures. This strong interaction of pores with water does not occur in traditional porous carbons even if they have high microporosity. In particular, GU13‐500 with higher pore volume shows the highest H_2_O uptake capacity of 425 cm^3^ g^−1^, corresponding to 19.4 mmol g^−1^ at 1 bar. In addition, no saturation was attained in the 1 bar pressure range, which means that a higher H_2_O adsorption capacity could be obtained with further increase in the pressure. In accordance to the nitrogen physisorption experiments, the GU13‐500 has a higher overall water uptake, owing to its higher pore volume, as compared to that of GU13‐350. The superior H_2_O adsorption ability for the C_2_N*_x_*O_1‐_ *_x_* indicates the presence of strong water‐binding sites in the C_2_N‐type structure, which is similar to those of the zeolites. Since this unusually high hydrophilicity is found in all the tested C_2_N configuration materials synthesized using different methods and precursors, the properties discussed could be considered to be inherent properties, and therefore have broad significance.

## 4 Characterization of C_2_N

Characterization of C_2_N is basically similar to that of porous polymers and carbons. Typical characterization includes studying the connectivity of atoms, structural regularity, morphology, and porosity. Fourier‐transform infrared spectroscopy (FTIR) is used to obtain the degree of condensation.^[^ ^57^ ^]^ Powder X‐ray diffraction (PXRD) is applied to analyze the structural regularity and the long range ordering.^[^ ^2^ ^]^ Elemental analysis was accomplished as combustion analysis to determine the elemental compositions.^[^ ^3^ ^]^ X‐ray photoelectron spectroscopy (XPS) is a surface analysis technique that provides chemical and electronic state information, making it suitable for characterizing and understanding the local bonding environment of chemical species.^[^ ^107^ ^]^ And thermogravimetric analysis (TGA) is carried out to provide first‐hand information to investigate the thermal stability of C_2_N. The C_2_N materials are really noble, and they are hard to oxidize and combust. The oxidation stability could be display in TGA test under air conditions.^[^ ^37^ ^]^ Scanning electron microscopy (SEM) and transmission electron microscopy (TEM) are used to analyze the morphology and the microstructure of the materials.^[^ ^38^ ^]^ High‐resolution transmission electron microscopy (HR‐TEM) and scanning tunneling microscope (STM) are used to further study properties of materials on atomic scale.^[^ ^2^ ^]^ The textural properties of the samples are usually assessed by nitrogen adsorption and desorption. However, it is widely known that nitrogen physisorption in strongly polarizing materials, like C_2_N, will be influenced by specific adsorbent‐adsorbate interactions resulting from the high quadrupole moment of nitrogen. Therefore, the micropore volumes and pore size distributions are checked by argon physisorption.^[^ ^7^ ^]^

In addition to experimental research, theoretical calculations are necessary and useful assistive techniques for studying the structure and function of a framework. Most theoretical research have focused on the structural modeling of C_2_N, while other theoretical studies have pointed out properties and applied predictions. Molecular modeling provides important information for predicting the characterization, properties, and applications of C_2_N,^[^ ^52^, ^53^ ^]^ and we foresee their importance in the future will increase.

## 5 C_2_N Applications

Owing to its distinctive physiochemical properties, including stronger polarized surfaces, increased hydrophilicity, higher ion binding, and thermal and chemical stability, a broad range of C_2_N applications have been explored. The unique pore structure tightly assembled with nitrogen atoms has been widely employed in gas adsorption, energy storage, and biomedical applications. Furthermore, the rich nitrogen content and N6‐cavities in C_2_N makes it suitable for anchoring metallic nanoparticles and single atoms, rendering it an “active” or “non‐innocent” support.

### 5.1 CO_2_ Adsorption and Separation

The large quantity of CO_2_ emitted by human civilization is responsible for climate change, sea level rise, or increased extreme weather events. The first necessary step to enter into a potential circular economy based on CO_2_ is to bind the CO_2_. As of today, there are various techniques for CO_2_ adsorption and separation. Among them, adsorption using porous materials is attractive because the process is clean and extremely efficient. The application of C_2_N, which have a high adsorption enthalpy for CO_2_, is quite promising, owing to its high porosity and multipodal binding sites.

As described above, the microporous carbons with C_2_N‐type stoichiometry obtained using direct carbonization of prealigned HAT condensates^[^ ^38^ ^]^ exhibited high specific surface areas of 1000 m^2^ g^−1^, high micropore volume, and high stability. At condensation temperatures of 525 °C and below, the adsorption of nitrogen gas was negligible, but the internal pores were large enough to allow CO_2_ to be adsorbed inside the structure due to size exclusion, leading to a molecular sieving effect. This resulted in surprisingly high CO_2_ adsorption capacities and isosteric heat of adsorptions of up to 52 kJ mol^−1^. This adsorption energy was high enough to be active in a complete ambient temperature range and exceeded the values for that of apolar gases like N_2_ significantly; hence, we predicted that they were also suitable to be used with gas streams where CO_2_ was highly diluted. A strong exothermic character also proved that this was an adsorption process and not absorption, i.e., Henry‐law like dissolution, where the enthalpy is much less. Theoretical calculations indicated that such a high binding enthalpy resulted due to the electron acceptor property of the carbon atoms in C_2_N, which were in close proximity to the oxygen atoms in CO_2_, and collective rebinding/stabilization effects of the nitrogen atoms in the C_2_N layers surrounding the carbon atom of CO_2_.^[^ ^108^ ^]^

This unusually high performance in CO_2_ adsorption was also exhibited by O‐substituted or O and S‐co‐substituted C_2_N materials. The C_2_N*_x_*O_1‐_ *_x_* and C_2_(N*_x_*O*_y_*S*_z_*)_1_ materials reported by us have heterocyclic rings with approximately 30 wt% of heteroatoms.^[^ ^37^, ^40^ ^]^ The remarkably high content of heteroatoms can only be meaningfully assigned and included in the structure as phenazines, oxazine, dioxine rings, or thiazine/thiazole, which then define the pore surface. As a result, these materials also showed heat of adsorption values of ≈50 kJ mol^−1^, which promote rather selective sorption processes.^[^ ^37^ ^]^ Here, it is important to mention that the strength of the interaction between CO_2_ and C_2_N is far from being similar to that between CO_2_ and porous heteroatom doped carbons, where physisorption in the material pores is the main sorption mechanism (enthalpies oscillate between 20 and 30 kJ mol^−1^ for these materials).^[^ ^109^ ^]^

### 5.2 C_2_N as Electrode Material for Batteries

Lithium ion batteries are widely used owing to their low weight and high energy density. Graphite is regularly used as an anode host material, but it could be complemented with C_2_N materials for more versatile energy storage. Pyridinic nitrogen in the C_2_N pores can act as redox‐active centers and interact strongly with the metal ions. Xu et al. reported that layered 2D frameworks with well‐defined crystal structures are promising candidates for high‐performance rechargeable lithium‐ion batteries (LIBs).^[^ ^47^ ^]^ As anode materials for LIBs, a C_2_N‐450 material showed outstanding electrochemical properties, including high reversible capacities of 933.2 and 40.1 mAh g^−1^ at 0.1 and 10 C, respectively. This is approximately three times the value for graphite.

**Figure 7:**
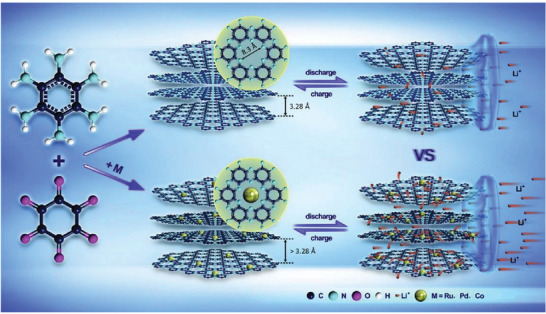
Scheme of the synthesis and lithiation–delithiation mechanism of M@C_2_N (M = Ru, Pd, and Co) and C_2_N. Reproduced with permission.^[^ ^110^ ^]^ Copyright 2018, Elsevier.

Introducing metal‐nitrogen active sites in C_2_N are beneficial for enlarging the distance between C_2_N nanosheets, which promotes the rapid diffusion and storage of Li^+^. Huang et al. reported a series of highly crystalline M@C_2_N hybrids in which the metals (M = Ru, Pd, and Co) were uniformly embedded in the 2D C_2_N networks (**Figure** **7**).^[^ ^110^ ^]^ In combination with the unique structural features of C_2_N, the lithium storage properties were determined as 1104.2, 1168.3, and 789.7 mAh g^−1^ for Ru@C_2_N, Pd@C_2_N, and Co@C_2_N at 0.1 C, respectively.

Doping sulfur into the C_2_N framework modulates the charge distribution and enables fast electron transfer, which promotes the formation of oxygen‐activating catalytic active sites and facilitates the use of C_2_N in advanced rechargeable metal‐air batteries. Shinde et al. reported a hierarchical 3D sulfur‐modulated holey C_2_N aerogel (S‐C_2_NA) that had an ultrahigh surface area (≈1943 m^2^ g^−1^) and exhibited excellent electrocatalytic activity; their lowest reversible oxygen electrode index of ≈0.65 V was superior to that of the highly active dual‐functional and commercial (Pt/C and RuO_2_) catalysts, i.e., the current gold standards. The favorable electronic structure and atomic coordination of the holey C–N skeleton enabled the reversibility of oxygen reactions. Using the materials as S‐C_2_NA air cathodes for Zn–air batteries with liquid electrolytes and solid‐state batteries exhibited excellent energy densities (958 and 862 Wh kg^−1^), low charge‐discharge polarizations, excellent reversibility, and ultralong cycling lives (750 and 460 h) as compared to those of the commercial Pt/C+RuO_2_ catalysts. Li‐O_2_ batteries with S‐C_2_NA demonstrated an outstanding specific capacity of ≈648.7 mAh g^−1^ and reversible charge–discharge potentials over 200 cycles.^[^ ^48^ ^]^

The transformation of bulk C_2_N into 0D quantum dots (QDs) may cause unique quantum confinement and edge effects, resulting in improved performance. Hu et al. reported that the synthesis of water‐soluble C_2_NQDs with an average size of less than 5 nm gives active oxygen containing edges, which creates an interesting multifunctionality. To modify commercial separators, the C_2_NQDs are introduced as new metal‐free catalysts to enhance polysulfide redox kinetics and give Li–S batteries great cycling stability, high rate performance, and larger areal capacity (7.0 mAh cm^−2^) under a high sulfur loading of 8.0 mg cm^−2^. These results indicated that the oxygenated edges enriched in the C_2_NQDs significantly improved the polysulfide immobilization and catalytic conversion.^[^ ^111^ ^]^

These results in general illustrated great potential of the C_2_Ns for diverse next‐generation battery devices.

### 5.3 C_2_N as Electrode Material for Supercapacitors

Supercapacitors have excellent power density, fast charging/discharging rates, and a long life, and they represent an emerging class of power sources. Carbon materials are a standard choice for electrodes in supercapacitors owing to their large specific surface area and high conductivity. Doping of N, O, and S and pore engineering have proven to be effective strategies in improving the capacitive performance of carbon materials. Moreover, in this context, an extension to much higher heteroatom contents looks promising.

We prepared C_2_(N*_x_*O*_y_*S*_z_*)_1_ that exhibited an extremely high N/O/S heteroatom content (<33 mol%) and large specific surface area (1704 m² g^−1^).^[^ ^40^ ^]^ In particular, the high sulfur content that can be maintained at relatively high temperatures is significant and unusual for traditional microporous carbons. Owing to the porosity of the structure and polarizability controlled by heteroatoms, the C_2_(N*_x_*O*_y_*S*_z_*)_1_ obtained at 800 °C attained a record high capacitance of 255 F g^−1^ at 3.5 V when used in an ionic liquid (IL) base supercapacitor cell. This remarkably high specific capacitance resulted due to the strong binding of electrolyte ions on the surface of the strongly polarizing material. In addition, as described for carbons by Simon and Gogotsi, their well‐developed micropores interacted strongly with single electrolyte ions, effectively promoting and addressing this “desolvation part” of energy storage.^[^ ^112^ ^]^ In contrast, the uniform mesoporosity acted as transport channels for ion diffusion.

A hybrid‐ion capacitor represents an innovative candidate for electrochemical energy storage because it combines the advantages of high‐power electrochemical capacitors and high energy batteries. Owing to the strong interaction of metal ions and electrolyte constituents of nitrogen‐containing functionalities in the well‐defined micropores, C_2_N exhibit superior performance in electrochemical energy storage to that of nitrogen‐free pristine carbons. However, their semiconducting character makes their conductivity lower than that of bare carbons. Thus, C_2_N/carbon composites are also a strategy to improve energy storage performance, providing abundant active sites through C_2_N and high electrical conductivity through pristine carbon materials.

**Figure 8:**
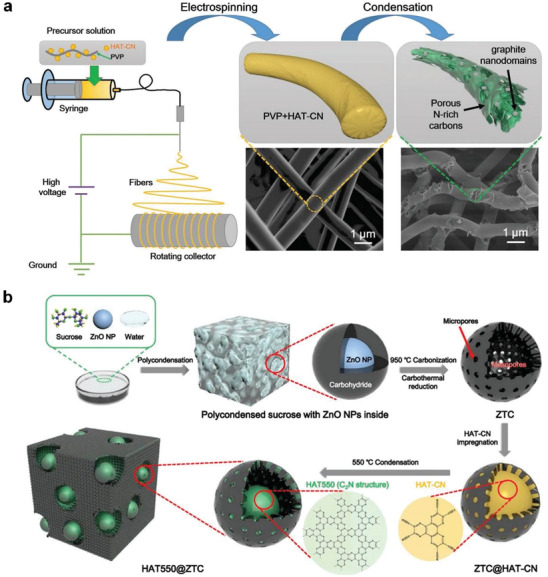
a) Preparation of porous HAT‐CNF using electrospinning followed by condensation. Reproduced with permission.^[^ ^45^ ^]^ Copyright 2019, Elsevier. b) Preparation of HAT550@ZTC. Reproduced with permission.^[^ ^46^ ^]^ Copyright 2020, Elsevier.

Yan et al. electrospun a mixture of polyvinylpyrrolidone and hexaazatriphenylene‐hexacarbonitrile (HAT‐CN) and produced microporous nitrogen‐rich carbon fibers (HAT‐CNFs) by subsequent thermal condensation (**Figure** **8**a).^[^ ^45^ ^]^ They adjusted bonding motives, nitrogen heteroatoms content, electronic structure, porosity, and degree of carbon stacking by modifying the condensation temperature. The obtained HAT‐CNFs show significant reversible capacities (395 mAh g^−1^ at 0.1 A g^−1^) and rate capabilities (106 mAh g^−1^ at 10 A g^−1^) as sodium storage anode materials owing to a combination of high heteroatoms content, enhanced electrical conductivity, and fast charge carrier transport in the nanoporous structure of the 1D fibers. Later they reported a nanocomposite material composed of C_2_N nanoparticles embedded in a conductive mesoporous carbon matrix (Figure 8b).^[^ ^46^ ^]^ Using the synergistic effect of hybridizing C_2_N material (with high N heteroatom content) and conductive carbon (with fast electron storage and transport abilities), superior capacity and rate capabilities were achieved in sodium storage. Such results proved that the C_2_N skeleton and pores in the composites could provide a strong interaction between the metal ions, which is crucial for the electrochemical capacity.

### 5.4 C_2_N in Catalysis

**Figure 9:**
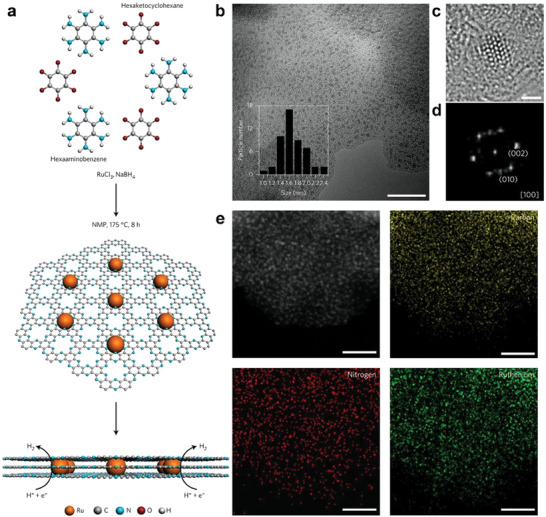
a) Schematic illustration of the synthesis and structure of Ru@C_2_N electrocatalyst. Hexaaminobenzene and hexaketocyclohexane in N‐methyl‐2‐pyrrolidone (NMP) react to form the C_2_N framework, while RuCl_3_ and NaBH _4_serve as the Ru precursor and reducing agent. b) TEM image of Ru@C_2_N. Inset: corresponding particle size distribution of the Ru nanoparticles. Scale bar: 20 nm. Atomic‐resolution TEM image of Ru@C_2_ Nc) and corresponding fast‐Fourier transform (FFT) pattern d) Scale bar: 1 nm. e) STEM image and STEM‐EDS element mapping of Ru@C_2_N. Scale bar: 20 nm. Reproduced with permission.^[^ ^39^ ^]^ Copyright 2017, Springer Nature.

As described above, the nitrogen‐lined pores of C_2_N provide a chemically active site for anchoring the metal nanoparticles/atoms. Its uniform pore structure provides an ideal support for chemical bonding between the metal nanoparticles/atoms and support. Moreover, a high specific surface area ensures sufficient number and exposure of the metal sites upon adsorption and catalysis of reactive molecules. Mahmood et al. reported a Ru‐based catalyst for the hydrogen evolution reaction (HER), which can operate both in alkaline and acidic media (**Figure** **9**).^[^ ^39^ ^]^ The catalyst Ru@C_2_N is made of Ru nanoparticles dispersed on top of the nitrogenated holey 2D C_2_N layers. Ru@C_2_N displays high turnover frequencies at 25 mV (0.75 H_2_ s^−1^ in 1.0 m KOH solution; 0.67 H_2_ s^−1^ in 0.5 m H_2_SO_4_ solution), small overpotentials at 10 mA cm^−2^ (17.0 mV in 1.0 m KOH solution; 13.5 mV in 0.5 m H_2_SO_4_ solution), and remarkable stability in both alkaline and acidic media. These performances are comparable and even better than those of a reference Pt/C catalyst for HER.

Qin et al. showed that Au single sites decorated on C_2_N‐type porous carbon catalysts exhibiting high performance in N_2_ electroreduction.^[^ ^51^ ^]^ A stable NH_3_ yield of 2.32 µg h^−1^ cm^−2^ is generated with a Faradaic efficiency of 12.3% (at a potential of 0.2 V vs reversible hydrogen electrode). Mahmood et al. reported the preparation of a C_2_N‐polymer‐encapsulated cobalt‐oxide (Co@C_2_N) catalyst using in situ solvothermal synthesis. The Co@C_2_N has excellent catalytic activities for hydrogen generation due to the alkaline hydrolysis of sodium borohydride (NaBH_4_). The rate of maximum hydrogen generation is comparable to the best reported values for catalysts containing other noble metals in alkaline solutions. In addition, the Co@C_2_N can also catalyze an in‐situ reduction of 4‐nitrophenol to 4‐aminophenol in the presence of NaBH_4_.^[^ ^50^ ^]^

**Figure 10:**
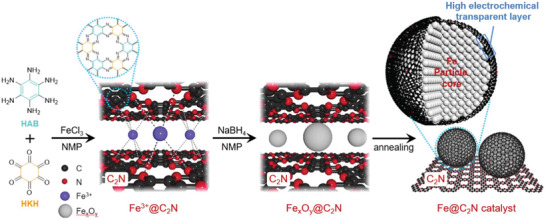
Schematic representation of the structural evolution of Fe@C_2_N catalyst, showing an in situ sandwiching of Fe^3+^in C_2_N layers (Fe^3+^@C_2_N) in NMP, reduction of Fe^3+^@C_2_N into Fe*_x_*O*_y_*@C_2_N by sodium borohydride, and subsequent annealing of Fe*_x_*O*_y_*@C_2_N into the Fe@C_2_N catalyst at 800 °C. The structure of the Fe@C_2_N catalyst consists of Fe nanoparticle cores encased in well‐ordered nitrogenated graphitic shells (Fe@C_2_N nanoparticles), which are uniformly distributed on the C_2_N matrix. Reproduced with permission.^[^ ^49^ ^]^ Copyright 2018, Elsevier.

The transformation of the otherwise unstable group VIIIB of transition metals into stable catalysts with a high performance in oxygen reduction reaction (ORR) remains a key challenge for electrochemical technologies. Iron‐nitrogen‐carbon‐based electrocatalysts have recently demonstrated ORR performances comparable to that of platinum‐based catalysts. However, their poor stability is still a key issue to be addressed. Mahmood et al. reported a catalyst obtained using an in situ sandwiching of a Fe^3+^ precursor in the C_2_N network (**Figure** **10**). The reduction of the sandwiched Fe^3+^ leads to the formation of iron oxide (Fe*_x_*O*_y_*) nanoparticles. Subsequently, annealing gives highly crystalline cores of Fe^0^ nanoparticle, while the C_2_N transforms into a well‐defined, encapsulating, and nitrogenated graphitic shell during the heat treatment. The obtained Fe nanoparticles are uniformly distributed on the substrate and exhibited ORR activities superior to those of the commercial Pt/C catalysts in both acidic and alkaline media. In addition, even after 650 h, the Fe@C_2_N catalyst remained stable during harsh electrochemical testing, suggesting an exceptional durability.^[^ ^49^ ^]^

### 5.5 C_2_N for Biomedical Application

**Figure 11:**
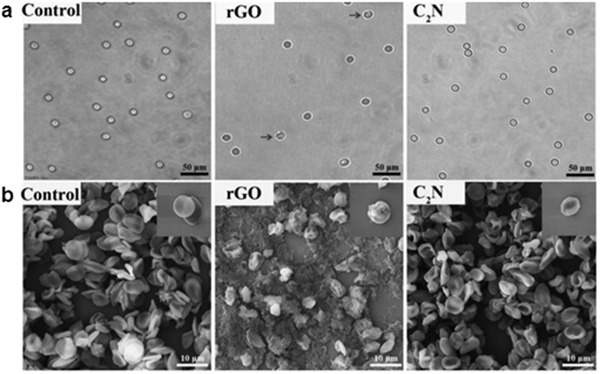
a) Optical microscopy and b) SEM images of red blood cells (RBCs) treated by rGO and C_2_N at 200 µg mL^−1^. Diluted RBCs incubated in 1 × PBS were regarded as negative control. The black arrows in panel A indicate lysed RBCs. Reproduced with permission.^[^ ^113^ ^]^ Copyright 2018, Wiley‐VCH.

The extensive use of nanomaterials (e.g., carbon‐based 2D nanomaterials) in biomedical applications, relies on their biocompatibility, particularly how they may affect the integrity of the cell membranes or disrupt endocrine chains or DNA processes. Liu et al. used a combination of experimental and theoretical approach to explore the interactions between C_2_N and human red blood cell membranes.^[^ ^113^ ^]^ In comparison with the control system (i.e., reduced graphene oxide‐rGO), the microscopic experiments display that C_2_N has negligible hemolysis effect on the red blood cells (RBCs) and excellent compatibility with the cell membranes (**Figure** **11**). Molecular dynamics simulations reveal further details on the potential molecular mechanism, which indicate that C_2_N prefers to be adsorbed on the water–membrane interface rather than disrupting the membrane; this is in good agreement with its higher hydrophilicity. Interaction energy analyses illustrate the key role of Columbic contributions, which stems from the unique electrostatic potential surface of C_2_N, in preventing C_2_N from penetrating into the cell membranes. Zhang et al. then systematically investigated the potential cytotoxicity of C_2_N nanosheets. In comparison with graphene oxide, C_2_N has a relatively mild cytotoxicity. Notably, this novel material exhibits negligible disruption of cell membranes, suggesting that C_2_N might be a potential alternative to graphene and its derivatives in biomedical research.^[^ ^114^ ^]^

C_3_N porous frameworks exhibited similar results.^[^ ^7^ ^]^ The samples prepared using gallic acid and 2,3‐diaminophenazine at 500 °C in the presence of salt melts show well‐defined porous structures and strongly polarized pore walls. As a result, the loaded drug molecules (e.g., aspirin) are unable to crystallize due to quantum confinement and strong interaction with the pore wall surfaces. Such strong interactions translated into lower melting points of the guest drugs. Remarkably, the guest drugs are released much faster than the crystalline ones.

## 6 Summary and Perspectives

The pore structure and pore surface chemistry are used to determine the properties and applications of porous materials. C_2_N offers a fine combination of well‐defined pore size, which is in the medium micropore range, and an extremely well‐controlled surface chemistry that exhibits properties such as high nobility, high surface area with multipodal binding sites, and high hydrophilicity. C_2_N can be synthesized via three methods: wet‐chemical condensation, salt melt synthesis, or direct carbonization of suitable monomers. The C_2_N materials have performed excellently in a variety of applications (gas adsorption and separation, batteries, supercapacitors, catalysis and biomedicine, etc.). These unique properties can be attributed to the following: 1) the well‐defined structural pores and strongly polarizable pore surface generate size sieving and surface binding effects that enhances its performance in gas adsorption and separation; 2) C_2_N with inherent open nanopores and additional hierarchical porosity promotes the transport of electrolyte and metal ions and provides multiple active affinity sites, thereby greatly improving electrochemical energy storage performance; and 3) C_2_N can not only provide active centers and sites to catalyze diverse reactions by itself, but also effectively stabilize metal atoms/nanoparticles owing to the transfer of electron density when used as an active catalytic support. In addition, the highly dispersed metal states are stabilized effectively. Owing to these characteristics, C_2_N has a great potential in other fields such as biological applications.

Although the initial years of C_2_N study offered a great deal of surprises and performance records, several challenges are yet to be addressed. For example, most syntheses start—directly or indirectly—from a single, rather expensive monomer, hexaketocyclohexane. Low‐cost sustainable starting monomers and simplified reaction procedures, which allow a scaled production, are required to apply C_2_N in practical engineering applications. As C_2_N (or heteroatom‐substituted variants) seems to have a low free energy of formation, we expect some cascade reactions based on simple urea or aminonitriles to aid in this regard. Another question is the importance or role of pore order in such materials. In general, a crystalline mesoscale order is preferred when compared to a weakly ordered polymer state. In several disciplines as polymer science, catalysis, or metal research, the controlled disorder or grain boundaries contribute essentially to the properties. Therefore, the answer to the aforementioned question seems to depend on the property that scientists desire to optimize.

Furthermore, a deeper theoretical or modeling understanding of the relationship between the structure and performance of C_2_N is essential. For example, the synergistic mechanism of N6‐cavities and their communication via the paraconnection motifs need to be elucidated further, as this has a high relevance in metal loading. The effect of different heteroatom exchanges on the microstructure and properties of materials should also be studied further. The theoretical calculations have proven to be useful in understanding the interaction between C_2_N pores and guest molecules, and the applicability of C_2_N in some of these new fields has been predicted by a number of modeling groups. However, experimentally, the scientists are still far from achieving some of these results in their laboratories.

A single metal atom catalyst using C_2_N as a noninnocent support is another groundbreaking innovation. Their interaction potentials have been calculated to be approximately 500 kJ mol^‐1^, i.e., including the most stable chemical bonds; however, the question of the commensurability of orbital hybridization symmetry (e.g., octahedral) and the stacked sixfold planar symmetry of the solid state ligand was still not posed. To our expectation, this mutual optimization problem can result in new symmetry‐controlled electron transfer processes as well as new metal–ligand hybridization schemes.

In our opinion, there are several exciting possibilities for research in this field. In future, we will be focusing on analyzing the physical properties of water within those N6‐cavities using NMR and impedance spectroscopy, and to understand the physical state of hydrated ions located in these nanopores. In the longer run, we hope that such efforts can contribute to improving deionization membranes and water gating processes. Moreover, the coupling of water and electric transport across these (conducting) carbon materials is of key interest, such that the material could contribute to artificial ion pumps, blue energy generation, and controlled specific‐ion gating.^[^ ^115^ ^]^

Another unexplored field is the redox behavior of the pyrazinic centers (ordinary pyrazine can take up two protons and two electrons), which were partially instrumentalized in battery and supercapacitor anodes, but were kept out of systematic examinations. The electron density and functional groups of the product obtained could be carefully adjusted such that its electrochemical and catalytic capabilities are amplified or tuned for new application areas by controlling redox chemistry. In addition, the processing of such C_2_N into various forms (e.g., membranes) is an effective way to diversify its engineering applications. In this regard, the material may even be directionally grown on film substrates to use it as thin and defect‐poor versions of this controlled “per‐porated” type of graphene.

## Conflict of Interest

The authors declare no conflict of interest.
